# A de novo synonymous variant in *EFTUD2* disrupts normal splicing and causes mandibulofacial dysostosis with microcephaly: case report

**DOI:** 10.1186/s12881-020-01121-y

**Published:** 2020-09-17

**Authors:** Arthur Jacob, Jennifer Pasquier, Raphael Carapito, Frédéric Auradé, Anne Molitor, Philippe Froguel, Khalid Fakhro, Najeeb Halabi, Géraldine Viot, Seiamak Bahram, Arash Rafii

**Affiliations:** 1 Univ. Lille, CNRS, CHU Lille, Institut Pasteur de Lille, UMR 8199 – EGID, F-59000 Lille, France; 2 Stem Cell and Microenvironment Laboratory, Weill Cornell Medicine-Qatar, Education City, Qatar Foundation, Doha, Qatar; 3 Institut National de la Santé et de la Recherche Médicale (INSERM), Centre National de la Recherche Scientifique (CNRS), UMR_S 938, Centre de Recherche Saint-Antoine, Team Cancer Biology and Therapeutics, Institut Universitaire de Cancérologie, Sorbonne Université, F-75012 Paris, France; 4 Nice Breast institute, 57 bld de la Californie, 06000 Nice, France; 5 Laboratoire d’ImmunoRhumatologie Moléculaire, plateforme GENOMAX, INSERM UMR_S 1109, Faculté de Médecine, Fédération Hospitalo-Universitaire OMICARE, Fédération de Médecine Translationnelle de Strasbourg (FMTS), LabEx TRANSPLANTEX, Université de Strasbourg, 4 rue Kirschleger, 67085 Strasbourg, France; 6 INSERM IMRB U955-E10, UPEC - Université Paris Est, Faculté de Médicine, 94000 Créteil, France; 7 Department of Genomics of Common Disease, School of Public Health, Imperial College, South Kensington Campus, London, SW7 2AZ UK; 8 Epigenetics Cardiovascular Laboratory, Department of Genetic Medicine, Weill Cornell Medicine-Qatar, Doha, Qatar; 9 Department of Human Genetics, Sidra Medical and Research Center, Doha, Qatar; 10 Stem cell and microenvironment laboratory, Weill Cornell Medical College in Qatar, Education City, Qatar Foundation, Doha, Qatar; 11 Gynécologie Obstétrique, HUPC, Hôpital Cochin, HUPC, Assistance Publique - Hôpitaux de Paris, Paris, France; 12 Department Genetic Medicine, Weill Cornell Medical College, New York, NY USA

**Keywords:** *EFTUD2*, Mandibulofacial dysostosis with microcephaly, de novo, Synonymous splice variant, Exonic splice enhancer variant, Whole-exome sequencing, Case report

## Abstract

**Background:**

Mandibulofacial dysostosis with microcephaly (MFDM) is a rare autosomal dominant genetic disease characterized by intellectual and growth retardations, as well as major microcephaly, induced by missense and splice site variants or microdeletions in the *EFTUD2* gene.

**Case presentation:**

Here, we investigate the case of a young girl with symptoms of MFDM and a normal karyotype. Whole-exome sequencing of the family was performed to identify genetic alterations responsible for this phenotype. We identified a de novo synonymous variant in the *EFTUD2* gene. We demonstrated that this synonymous variant disrupts the donor splice-site in intron 9 resulting in the skipping of exon 9 and a frameshift that leads to a premature stop codon.

**Conclusions:**

We present the first case of MFDM caused by a synonymous variant disrupting the donor splice site, leading to exon skipping.

## Background

Mandibulofacial dysostosis with microcephaly (MFDM) is a rare autosomal dominant disease characterized by malar and mandibular hypoplasia and microcephaly. Some of its main features include conductive hearing loss, intellectual disability, distinctive facial features and craniofacial malformations that may include characteristic external ear malformations, cleft palate, choanal atresia, and facial asymmetry. In some instances, one observes extracranial malformations such as esophageal atresia (~ 40%), congenital heart disease (~ 40%), and thumb abnormalities (~ 25%). Short stature is present in approximately one-third of individuals [1–4].

Its exact prevalence is unknown, but more than 80 cases have been described in the literature until now. MFDM is mostly caused by de novo variants in the *EFTUD2* gene (MIM# 603892) [5]. In some rarer instances, the MFDM is transmitted from a parent in an autosomal dominant manner (19% of the cases) or due to germline mosaicism (6% of the cases). *EFTUD2* encodes the U5-116kD, a highly conserved GTPase component of the major spliceosome complex that processes precursor mRNAs to produce mature mRNAs by allowing the dissociation of U4 and U6 snRNPs during splicing in a GTP-dependent manner [6].

The *EFTUD2* gene is composed of 29 exons and presents four transcript variants encoding three different isoforms. Seventy-six distinct single-nucleotide variants (SNVs) and seven microdeletions in *EFTUD2* involved in MFDM have been described to date [5]. They can alter basic, surface-forming residues that are potentially available for protein-protein interactions in the internal face of the protein and could conceivably affect protein stability by several mechanisms acting on protein stability, conformation, localization, and/or post-translational modifications. Various types of *EFTUD2*-variants have been identified, including missense, frameshift, intronic splice site variants and deletions. However synonymous splice site variants in the gene have never been previously implicated in this disease.

Synonymous variants initially do not appear to alter the structure and function of the proteins. They have long been interpreted as “silent” variants. Studies in evolutionary genetics have, however, shown that not all synonymous codons are used at the same frequency in the genome and that selection pressure is exerted even on the synonymous codons as they are used differently for mRNA splicing, translation, and processing machinery. The association of synonymous variants with over 50 human diseases has further confirmed the importance of these phenomena [7].

## Case presentation

Here, we report a seven-year-old female patient, who is a native of Libya, who presents postnatal microcephaly to -3SD, sensorineural hearing loss, and global intellectual delay with difficulties of comprehension. She also presents epileptic seizures, livedo and facial dysmorphisms such as micro-retrognatism, malar hypoplasia, dental malocclusion, limitation of mouth opening, and large protruding ears.

As her karyotype was normal and her parents were both healthy, we performed whole-exome sequencing (WES) of the child and her parents to identify putative genetic alterations responsible for this phenotype. WES was performed on genomic DNA prepared from the patient and the parents’ blood samples. The mean coverage of the exome-wide regions was 139.09, 119.25, and 148.62 reads, corresponding to a coverage of at least 10 reads of 95.99, 95.91, and 96.08% of the exome for the patient, mother, and father, respectively. In our variant analysis, we prioritized variants that were rare in the healthy population according to GnomAD v3 database (< 1%), the variants predicted to be deleterious on protein function according to SIFT and PolyPhen tools, and transmitted as compound heterozygous or arose de novo, consistent with the non-consanguineous and healthy parent context (Table S1).

**Fig. 1:**
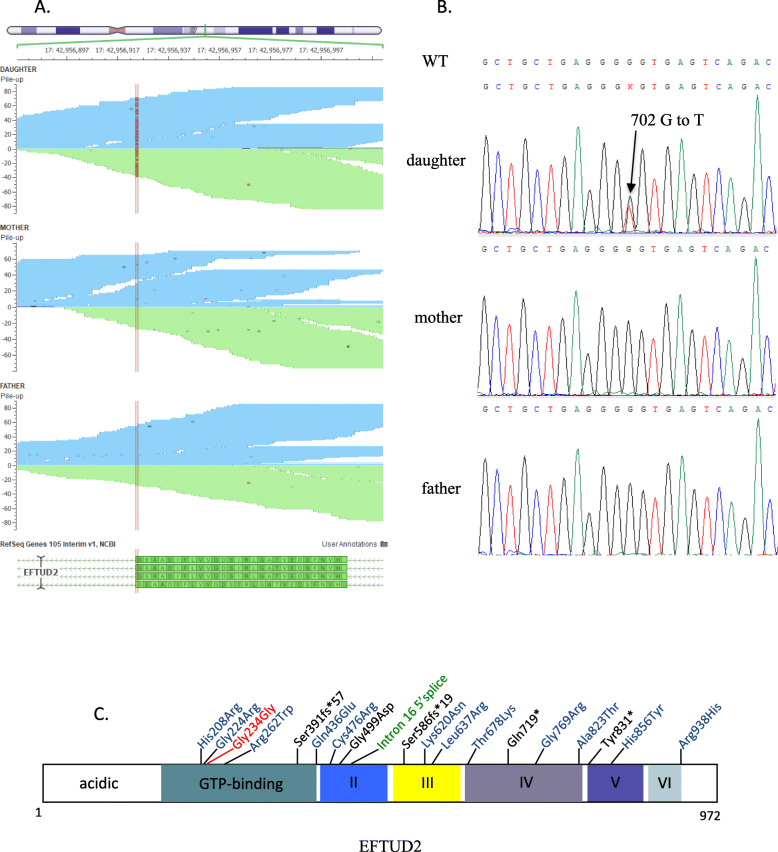
Identification of the de novo variant in the patient. **a** The graphs present the Whole Exome Sequencing pile-up reads of the region of interest. The red lines highlight the identified de novo NM_004247.4 c.702G > T variant in *EFTUD2* gene in the patient (Top graph) compared to its parent. **b** Electropherograms from Sanger sequencing of a nucleotide change from G to T in the proband (Daughter) compared to her parents. This variant is a heterozygous variant as both alleles harbor a different nucleotide. **c** Schematic view of *EFTUD2* protein structure, with the location of c.702G > T, p.Gly234Gly and of some other variants of different types causing MFDM disease. The synonymous variant identified in our patient is in red, missense mutations are shown in blue, truncating (nonsense and frameshift) mutations are shown in black, and the intron splice donor mutation is shown in green

Among these pertinent variants, the only one that could explain the patient’s phenotype was the de novo synonymous variant c.702G > T (transcript NM_004247.4) in the exon 9 of *EFTUD2* at position chr17:42956924 (GRCh37/hg19) in the patient (Fig. 1a)*.* This variant replaces a GGG codon to GGT, resulting in the retention of glycine at amino acid residue 234 (p.G234G). According to ACMG 2015 guidelines [8], this variant is classified as having unknown significance. Sanger sequencing confirmed that neither parents carried the variant (Fig. 1b). The variant is located in the G-domain of the protein, which is known to bind and hydrolyze GTP and a site of other variants of *EFTUD2* gene that are associated with MFDM (Fig. 1c)**.** As MFDM disease patterns seem to correspond closely to the symptoms of the patient (Table 1), we decided to investigate the potential impact of this synonymous variant on *EFTUD2* function.

**Table 1:** Detailed clinical features of our patient compared to the spectrum of clinical symptoms observed in patients with MFDM

Features	Our patient	All reported individuals	Estimated penetrance (%)
**Craniofacial**			
Micrognathia	Yes	87/89	98
Small or dysplastic pinna(e)	Yes	84/87	97
Malar hypoplasia	Yes	78/84	93
Hearing loss	Yes	69/83	83
Conductive	No	32/51	63
Mixed	No	13/51	25
Sensorineural	Yes	7/51	12
Auditory atresia /stenosis	No	47/73	64
Vestibular system abnormalities	No	14/25	56
Ossicular abnormalities	No	8/15	53
Facial asymmetry	No	25/47	53
Preauricular tag(s)	No	45/86	52
Cleft palate	No	41/88	47
Choanal atresia	No	27/83	33
Neonatal resuscitation	No	14/46	30
Tracheostomy	No	10/50	20
Limitation of mouth opening	Yes	7/85	8
**Extracranial**			
Thumb anomalies	No	24/77	31
Heart defects	No	28/89	31
Esophageal atresia	No	23/85	27
Renal malformation	No	9/85	10
**Development**			
Developmental delay	Yes	83/83	100
Microcephaly	Yes	78/89	88
Congenital	No	34/53	64
Postnatal	Yes	19/53	36
Epileptic seizures	Yes	21/77	27

**Fig. 2:**
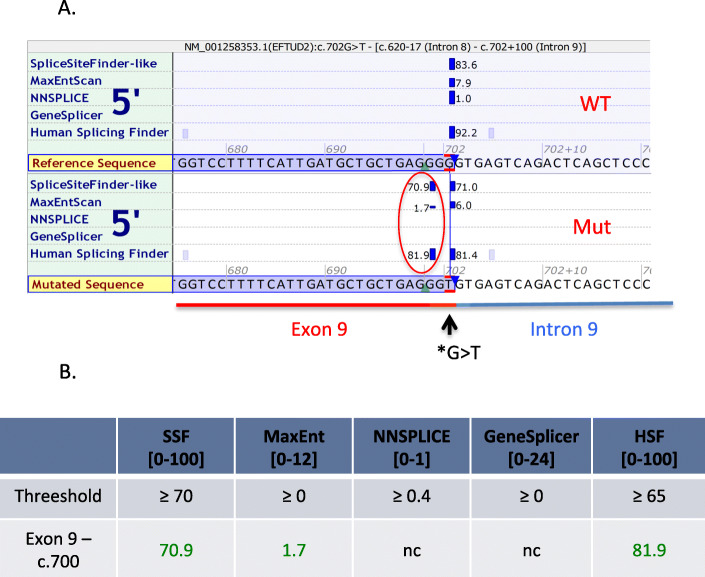
In silico predictions of the impact of NM_004247.4 (*EFTUD2*): c.702G > T variant on splicing. **a** Screenshot of in silico predictions of the impact of *EFTUD2* c.702G > T variant using Alamut software. Three out of five tools predicted that the G > T variant creates a new 5′ donor splicing site (red circle) in the mutated sequence (bottom rectangle) compared to wildtype sequence (top rectangle). The exon 9 (red) and intron 9 (blue) are highlighted on the Figure. **b** The table represents the splicing scores for the new 5′ donor splicing site by the five predicators tools. The threshold score to reach predicting the presence of a donor splice site is indicated for each predicator tool

The T allele at this position is novel in all public databases, including the NHLBI Exome Sequencing Project, the 1000 Genomes Project, and GnomAD v3, suggesting very high conservation of the G allele in the population. The mutated residue is the last nucleotide of exon 9, localized at the exon/intron junction adjacent to the splice donor site GT (c.702 + 1 and + 2). According to three splicing prediction tools - SpliceSiteFinder-like (SSF), MaxEntScore (MES) and Human Splicing Finder (HSF) - our variant affects the donor splice site by creating an alternative cryptic donor site “GT” preceding the original one (Fig. 2a, b).

**Fig. 3:**
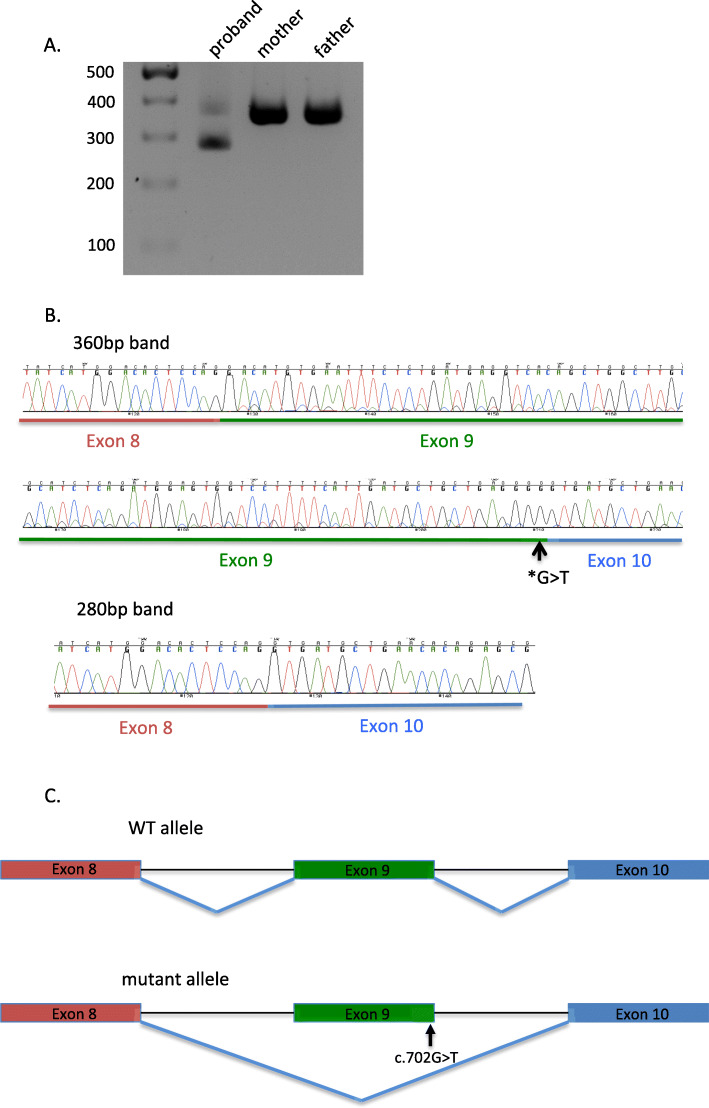
NM_004247.4 (*EFTUD2*): c.702G > T variant induces exon skipping. **a** Electrophoresis gel of *EFTUD2* cDNA obtained after amplification of the variant region from the proband and its parents. The proband displays two bands, one at 360 bp and one at 280 bp. **b** Electropherogram from Sanger sequencing 360 bp and 280 bp bands from the electrophoresis gel in A. The exon 8 is highlighted in red, the exon 9 in green and the exon 10 in blue. The exon 9 – exon 10 junction of *EFTUD2* cDNA shows exon 9 skipping in cDNA of 280 bp band which is presented only in the proband. **c** Schematic representation of the exon 9 skipping in the mutant allele of the proband compared to the wild type (WT) allele

To test the prediction, we investigated the consequence of the variant on the splicing of *EFTUD2* gene in vivo, in peripheral blood of the proband and her parents. After RNA isolation from leukocytes, we performed an RT-PCR and amplified 360 bases covering exon 8 to exon 12 of *EFTUD2* cDNA. We observed in all three individuals the expected PCR product band of ~ 360 bp and an additional PCR product of ~ 280 bp in the proband only (Fig. 3a). This result suggests deletion of about 80 bp in the patient’s *EFTUD2* cDNA.

The sequencing of the alternative cDNA showed complete deletion of exon 9 (Fig. 3b and c). As the exon 9 length is not a multiple of 3 (83 bp), its deletion would trigger a frameshift leading to a premature stop codon that truncates the protein c.620_702del, p.His209Aspfs*25 (Supplementary Fig. 1). This result demonstrates that the de novo synonymous variant identified in *EFTUD2* is responsible for the splicing defect leading to the skipping of exon 9, an exon that is present in all splice isoforms of *EFTUD2*.

### Patients

The patient was recruited at the “Unité de Diagnostic Prénatal - CPDP” of the American Hospital of Paris. The parents gave their signed informed consent for the clinical exome sequencing of their child and themselves.

### Whole exome sequencing

Genomic DNA was isolated from peripheral blood using standard protocols. Exome sequencing libraries were prepared with the TruSeq Exome Kit (Illumina, San Diego, CA, USA) following the manufacturer’s recommendations. Paired-end (2 × 75 bp) sequencing was performed on a NextSeq500 sequencer (Illumina, San Diego, CA, USA).

### Bioinformatic analysis

FastQ data were aligned to the GRCh37 (hg19) reference genome with bwa-0.7.12 [9], sorted and indexed with samtools-1.2 [10], deduplicated with PICARD-1.110, and base corrected and indel realigned with GATK-3.8 [11, 12]. Variant calling was done with GATK-3.8 HaplotypeCaller in GVCF ERC mode. Variants were called individually for each sample and then combined with GATK-3.8 GenotypeVCFs to produce a combined VCF. The combined VCF was then uploaded and analyzed with Ingenuity Variant Analysis software. Alignments were visualized with GenomeBrowse (Golden Helix - Massachusetts). FastQC-0.11.5 was used to calculate quality metrics for FastQ files and Qualimap-2.2.1 [13] was used to calculate coverage statistics using the truseq-exome-targeted-regions-manifest-v1–2.bed file. The reference file used for alignment and variant calling was human_g1k_v37.fasta which was provided with the GATK b37 resource bundle.

### RNA isolation and RT-PCR

Peripheral blood samples from the proband and her parents were used for the analyses in this study. Peripheral blood mononuclear cells were isolated by Ficoll-Paque™ density gradient centrifugation. After total RNA extraction using Trizol, Reverse-Transcription and PCR were performed as described in [14]. Forward and reverse primer sequences purchased from IDT were respectively: 5′ GTGGAATACATGCTTATTAATCCATTGACC 3′ and 5′ GAGCAAGAGAGAGGTGTAGGCATC 3′.

PCR products were analyzed on a 2% agarose gel as described in [14]. Finally, we used PCR clean-up gel extraction from Macherey-Nagel to isolate DNA bands from the agarose gel for sequencing.

### Sanger sequencing

The *EFTUD2* variant was validated using capillary Sanger sequencing. Briefly, a 262 bp DNA stretch of *EFTUD2* was amplified using the Expand Long Template PCR System (Roche, Meylan, France), following the manufacturer’s recommendations. The PCR primer pair was 5′-TTCAAGTTCTCTGGCTCCCA-3′ (forward) and 5′-CCCTCAGTTCACCCTACCAG-3′ (reverse). After purification with the Exostar kit (GE Healthcare, Little Chalfont, UK), PCR products were bi-directionally sequenced with the same primers using Big Dye Terminator Kit v3.1 (Life Technologies). Sequence reactions were run on an ABI PRISM 3730xl sequencer (Life Technologies).

## Discussion and conclusions

The increased access to next-generation sequencing for clinical purposes has allowed the identification of thousands of novel pathogenic variants in different individuals. One of the main challenges in clinical genetics is the interpretation of pathogenicity from a sea of variants that remain largely of unknown significance.

Synonymous variants are often interpreted by default as being silent and benign given their predicted null impact on the protein sequence. However, there is evidence for some synonymous SNVs to affect RNA splicing, expression, folding and ultimately function, and, in doing so, contribute to the pathophysiology of many diseases [15–17].

In this case study, we report a synonymous c.702G > T variant in the *EFTUD2* gene. This variant has not previously been reported in the literature and is absent from large population databases (GnomAD, 1000 Genomes); without further analysis, our initial classification would have been of uncertain significance. However, in silico analysis predicted the disruption of normal splice site, prompting in vitro investigation of its biological significance. The sequencing of the whole exome did not identify other deleterious variants that could be of clinical interest. Although we cannot exclude the presence of relevant deleterious variations in the non-coding regions, the strong correlation between the patient’s phenotype and the clinical consequence of heterozygous alteration of *EFTUD2* was sufficient to assume its implication in the disease.

The synonymous variant modifies the consensus sequence between exon 9 and intron 9 from GGG|gt to GGT|gt. In contrary to in silico prediction tools that predicted the creation of an additional GT donor site (Fig. 2), the study of cDNA from blood showed that this variant disrupts the recognition of the donor site by the splicing machinery and results in complete skipping of exon 9. This result could give a hint to the limitations of predictive splicing tools that do not predict the disruption of the splice site induced by this variant. Our study is the first description of synonymous SNV of *EFTUD2* in an MFDM patient. Studying cDNA from blood can have some limitations mostly if the gene of interest has different transcripts with a tissue-specific expression; however, we ensured that the *EFTUD2* gene is ubiquitously expressed and that the different transcripts do not present differences such as alternative splicing in the region of interest.

Some exonic regions are involved in splicing regulation in highly conserved sites called exonic splice enhancers (ESEs) [18]. In 80% of splicing consensus sites, the last nucleotide of the exon is a “G” which is highly important for the recognition by the splicing machinery [19]. Recently, Savisaar et al. showed that ESEs are under strong selection pressure at synonymous sites, suggesting that synonymous variants in these sites may be a common cause of single-locus genetic diseases [20]. A deleterious missense variant in the last G nucleotide resulting in exon skipping has already been reported in *BRCA1* in 2 patients who developed breast cancer at a young age [21] and in a patient with retinitis pigmentosa [22]. To our knowledge, our study is the first to report a deleterious synonymous variant in the final nucleotide of an exon that results in exon skipping.

In conclusion, synonymous variants should not be disregarded especially when they are predicted to affect splicing according to in silico tools. This study provides important evidence for the classification of such variants.

## Supplementary information

**Additional file 1: Supplementary Fig. 1.** mRNA sequence of the WT allele versus the mutant allele. The exon 9 skipping in mutant allele is predicted to cause a frameshift, leading to a premature codon stop. The exon 8 is in red, exon 9 in green and exon 10 in blue.

**Additional file 2:** **Table S1.** Number of prioritized variants during the WES data filtering analysis.

## Data Availability

The hg19/GRCh37 human reference genome (https://genome.ucsc.edu/cgi-bin/hgTracks?db=hg19&position=lastDbPos) was used as the reference dataset in this study. The reference sequence used for the validation of the G234G variant in EFTUD2 was obtained from NCBI Nucleotide using the accession number NM_004247.4. The variant reported in here is available in the Clinvar repository, with accession ID: SCV001251173. The datasets generated during the current study are not publicly available because it is possible that individual privacy could be compromised and the participants did not provide consent to make the data public.

## Abbreviations

ESE: Exonic Splice Enhancer
EFTUD2: Elongation Factor Tu GTP Binding Domain Containing 2
HSF: Human Splicing Finder
MES: MaxEntScore
MFDM: Mandibulo Facial Dysostosis with Microcephaly
SNV: Single Nucleotide Variant
SSF: SpliceSiteFinder-like
WES: Whole Exome Sequencing
