# Supplementary material for: A de novo synonymous variant in EFTUD2 disrupts normal splicing and causes mandibulofacial dysostosis with microcephaly: case report
Source: BMC Med Genet. 2020 Sep 17;21:182. doi: 10.1186/s12881-020-01121-y (PMC7499997; doi:10.1186/s12881-020-01121-y)
Supplement: Supplementary file 1 — Additional file 1: Supplementary Fig. 1. mRNA sequence of the WT allele versus the mutant allele. The exon 9 skipping in mutant allele is predicted to cause a frameshift, leading to a premature codon stop. The exon 8 is in red, exon 9 in green and exon 10 in blue. [file 12881_2020_1121_MOESM1_ESM.pdf]

Supplementary Figure 1

WT allele

aaa	tct	aaa	tct	tat	ctc	ttc	aat	atc	atg	gac	act	cca	gga
K195	G196	K197	S198	Y199	L200	F201	N202	I203	M204	D205	T206	P207	G208
cat	gtg	aat	ttc	tct	gat	gag	gtc	aca	gct	ggc	ttg	atc	tca
H209	V210	N211	F212	S213	D214	E215	V216	T217	A218	G219	L220	I221	S222
gat	gga	gtg	gtc	ctt	ttc	att	gat	gct	gct	gag	ggg	gtg	atg
D223	G224	V225	V226	L227	F228	I229	D230	A231	A232	E233	G234	V235	M236
ctg	aac	aca	gag										
L237	N238	T239	E240										

Mutant allele

aaa	tct	aaa	tct	tat	ctc	ttc	aat	atc	atg	gac	act	cca	ggt
K195	G196	K197	S198	Y199	L200	F201	N202	I203	M204	D205	T206	P207	G208
gat	gct	gaa	cac	aga	gcg	gct	gat	caa	gca	tgc	ggt	gca	gga
D209	A210	E211	H212	R213	A214	A215	D216	Q217	A218	C219	G220	A221	G222
gag	gct	ggc	agt	cac	tgt	gtg	cat	caa	caa	gat	**tga**		
E223	A224	G225	S226	H227	C228	V229	H230	Q231	Q232	D233	**STOP**		

Legend : Exon 8 Exon 9 Exon 10
