# Supplementary material for: A de novo synonymous variant in EFTUD2 disrupts normal splicing and causes mandibulofacial dysostosis with microcephaly: case report
Source: BMC Med Genet. 2020 Sep 17;21:182. doi: 10.1186/s12881-020-01121-y (PMC7499997; doi:10.1186/s12881-020-01121-y)
Supplement: Supplementary file 2 — Additional file 2: Table S1. Number of prioritized variants during the WES data filtering analysis. [file 12881_2020_1121_MOESM2_ESM.docx]

Table S1: Number of prioritized variants during the WES data filtering analysis

| **Types of variants** | **Number of variants** |
| --- | --- |
| Exonic or splice sites variants | 11 258 |
| Rare variants (<1% in healthy population) | 1148 |
| Predicted deleterious variants (SIFT and PolyPhen) | 111 |
| Predicted deleterious *de novo* variants | 3 |
| Predicted deleterious compound heterozygous variants | 0 |
